# Evaluation of organotin (IV) dithiocarbamate compounds as potential anti-leukemia agents towards CCRF-CEM (CCL-119) cell line: Cytotoxic, apoptotic, cell cycle and genotoxic study

**DOI:** 10.1371/journal.pone.0329860

**Published:** 2025-08-11

**Authors:** Nabiha Hidayah Abdul Razak, Nur Rasyiqin Rasli, Norsyahira Mohamad Zamri, Asmah Hamid, Normah Awang, Nurul Farahana Kamaludin

**Affiliations:** 1 Program of Biomedical Science, Center for Toxicology & Health Risk Study (CORE), Faculty of Health Sciences, Universiti Kebangsaan Malaysia, Kuala Lumpur, Malaysia; 2 Environmental Health & Industrial Safety Program, Center for Toxicology & Health Risk Study (CORE), Faculty of Health Sciences, Universiti Kebangsaan Malaysia, Kuala Lumpur, Malaysia; Laurentian University, CANADA

## Abstract

Acute lymphoblastic leukemia (ALL) is a common cancer affecting children worldwide, and current treatment has adverse effects such as neurotoxicity. To overcome this problem, new organotin (IV) dithiocarbamate compounds were synthesized. In this study, the T-lymphoblastic leukemia cell line (CCL-119) was tested against seven new compounds: diphenyltin (IV) diisopropyl dithiocarbamate (ODTC 1), diphenyltin (IV) diallyl dithiocarbamate (ODTC 2), triphenyltin (IV) diisopropyl dithiocarbamate (ODTC 3), triphenyltin (IV) diallyl dithiocarbamate (ODTC 4), triphenyltin (IV) diethyl dithiocarbamate (ODTC 5), dimethyltin (IV) diisopropyl dithiocarbamate (ODTC 6) and dimethyltin (IV) diethyl dithiocarbamate (ODTC 7) hereafter referred to as ODTC 1–7, to identify their cytotoxic effects (MTT assay), mode of cell death (Annexin V FITC/PI staining) and effects on the cell cycle (RNase/PI staining) as well as genotoxic effects (alkaline comet assay). Results obtained after 24 hours of exposure showed that all organotin (IV) dithiocarbamate compounds (ODTC 1–7) exhibited potent cytotoxicity, with median inhibitory concentration (IC_50_) values ranging from 0.18 µM to 3.10 µM. The selectivity index showed that diphenyltin (IV) and triphenyltin (IV) dithiocarbamate compounds are more selective towards CCL-119 cells. All ODTC compounds induced apoptosis in CCL-119 cells, and each compound caused cell cycle arrest at specific phases, indicating diverse mechanisms of action. Apoptosis and cell cycle arrest were confirmed by flow cytometry. Treatment of the compounds caused significant (p < 0.05) DNA damage compared to the negative control, with ODTC 1 causing the highest genotoxic effects. In conclusion, diphenyltin (IV) and triphenyltin (IV) dithiocarbamate compounds show good potential as anti-leukemia agents. However, further studies on the mechanisms of action need to be conducted to have better insights into the effect of this compound on CCL-119 leukemia cells.

## 1. Introduction

Cancer is the most frequently diagnosed and one of the main causes of death. Based on the Malaysian National Cancer Registry Report [1], 10 cancers frequently occurred among Malaysians from 2012 to 2016. This includes leukemia, breast cancer, and lung cancer. Leukemia ranks among the most common cancers in Malaysia. It is a blood cancer that causes abnormal and immature blood cells. Generally, it affects the production of white blood cells. The production of white blood cells is abnormal and cannot function well to prevent and control any infections in the body [2]. In addition, leukemia cases also received attention in other countries. According to the American Cancer Society (2022) [3], 60650 cases of leukemia were newly diagnosed in 2022. There are four types of leukemia: acute lymphoblastic leukemia, acute myelogenous leukemia, chronic lymphoblastic leukemia, and chronic myelogenous leukemia. Acute lymphoblastic leukemia (ALL) often occurs in children. According to Terwilliger and Abdul-Hay (2017) [4], 80% of ALL cases involve children. Various efforts are being made to find a cure for this cancer. This includes chemotherapy treatment, radiation, transplantation, and immunotherapy [5]. The use of synthetic drugs in chemotherapy treatment is one of the useful treatments to treat ALL patients. However, this kind of treatment could lead to toxic side effects such as neurotoxicity and resistance towards the chemotherapeutic drugs [6]. To overcome this problem, new chemotherapeutic drugs are needed to reduce the side effects and drug resistance to achieve the best way to kill the cancer.

Nowadays, research on synthetic metal-containing anticancer agents is getting more attention as an anticancer agent. This is due to the potent cytotoxic effect on cancer cells. One of the new synthetic metal-containing anticancer or new synthetic compounds that was being studied was organotin (IV). Organotin (IV) is a compound that has tin (stannum) linked to the hydrocarbon. Previous studies showed that organotin compounds are very effective as an anticancer against various kinds of cancer [7]. In addition, the dithiocarbamate compound is a stable ligand that is strongly linked to the organotin (IV) and will enhance the effectiveness of the new synthetic organotin (IV) dithiocarbamate as an anticancer. In vitro studies of organotin (IV) dithiocarbamate have proved its effectiveness in killing cancer cells through its diversity of biological activities [6]. However, the potential of this organotin compound has not been studied thoroughly. Thus, in this study, we assessed the cytotoxic effects, modes of cell death, cell cycle analysis, and DNA damage induced by various organotin (IV) dithiocarbamate compounds in acute lymphoblastic leukemia CCL-119 cells. To comprehensively evaluate these biological responses, we employed a combination of well-established in vitro assays. The MTT assay was used to determine cell viability, as it reliably reflects mitochondrial metabolic activity and general cytotoxicity. Annexin V- FITC/PI staining enabled sensitive detection of early and late apoptosis by targeting phosphatidylserine externalization and membrane integrity. Cell cycle distribution was analyzed using PI/RNase staining, which provides accurate quantification of DNA content across cell cycle phases and identifies potential arrest points. Additionally, the alkaline comet assay was performed to detect DNA strand breaks at the single-cell level, offering high sensitivity in assessing compound-induced genotoxic stress. Collectively, these complementary assays were selected for their specificity, sensitivity, and relevance in addressing the underlying mechanisms of cell death and DNA damage. In designing the organotin (IV) dithiocarbamate compounds used in this study, specific functional groups such as methyl, phenyl, allyl, isopropyl, and ethyl were selected to evaluate how structural variations influence biological activity. These groups were chosen based on their distinct electronic and steric properties, which are known to affect compound reactivity, membrane permeability, and target binding. For instance, allyl groups contain reactive double bonds that can enhance interaction with biomolecules, isopropyl groups provide steric bulk that may influence selectivity, and ethyl groups contribute to lipophilicity and influence electron distribution around the tin center. These characteristics are believed to modulate cytotoxic and apoptotic potential, as supported by previous studies on organotin derivatives [6,8,9]

## 2. Materials and methods

### 2.1. Cell lines and culture conditions

The T-acute lymphoblastic leukemia cell line, CCRF-CEM (CCL-119™), and a non-cancerous cell, WIL2-NS (CRL-8155™), were purchased from the American Type Culture Collection (ATCC). Both are suspension types of cells and were cultured in Roswell Park Memorial Institute Medium 1640 (RPMI-1640) (Sigma-Aldrich), supplemented with 10% Fetal Bovine Serum (FBS) (Capricorn Scientific) and 1% penicillin-streptomycin (Nacalai Tesque) to obtain the complete growth medium and were incubated at 37⁰C with a 5% CO_2_ incubator. Both suspension cells are used for tests when the cells’ passage becomes passage 3–10 (maximum).

### 2.2. Compounds

**Fig 1:**
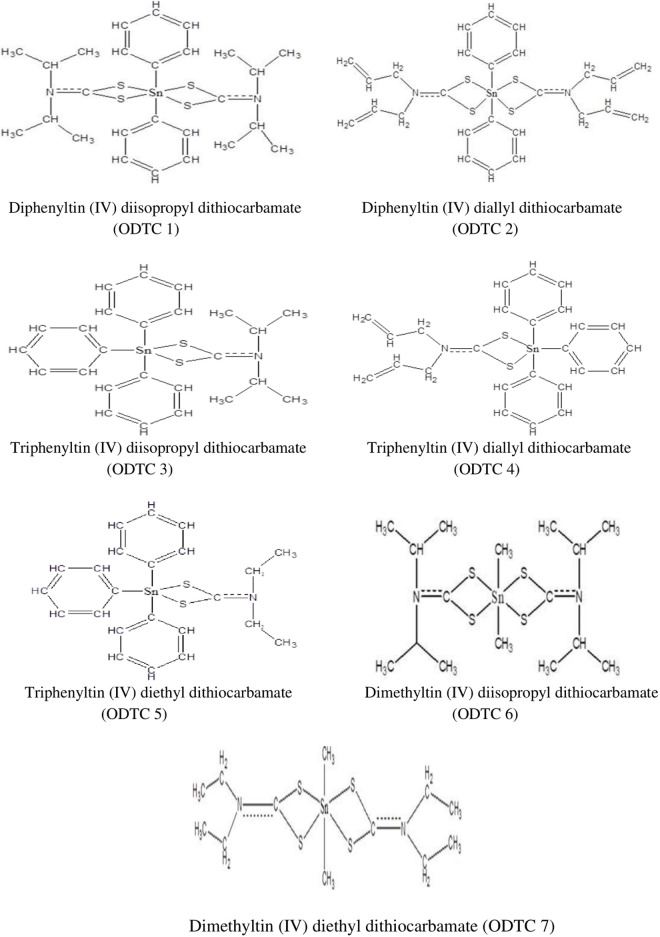
Chemical structure of organotin (IV) dithiocarbamate compounds.

Seven organotin (IV) dithiocarbamate compounds: diphenyltin (IV) diisopropyl dithiocarbamate (ODTC 1), diphenyltin (IV) diallyl dithiocarbamate (ODTC 2), triphenyltin (IV) diisopropyl dithiocarbamate (ODTC 3), triphenyltin (IV) diallyl dithiocarbamate (ODTC 4), triphenyltin (IV) diethyl dithiocarbamate (ODTC 5), dimethyltin (IV) diisopropyl dithiocarbamate (ODTC 6) and dimethyltin (IV) diethyl dithiocarbamate (ODTC 7) were synthesized and characterized by our team [9–12] at the School of Chemical Sciences and Food Technology, Faculty of Science and Technology, Universiti Kebangsaan Malaysia, Bangi. The chemical structure of seven different organotin (IV) dithiocarbamate compounds is shown in Fig 1 below.

### 2.3. Stock preparation

All stock solutions of the organotin (IV) dithiocarbamate compounds were prepared at 10 mM and dissolved in dimethyl sulphoxide (DMSO) (ChemAR®). The stock solutions were stored at 4°C; fresh dilutions were made by adding the RPMI-1640 medium before the experiment. The dilution is carried out using the serial dilution method to obtain the desired concentrations, which are 5 µM, 2.5 µM, 1.25 µM, 0.63 µM, 0.32 µM, and 0.16 µM in this study. Vincristine (VCR) is used as a positive control in this study.

### 2.4. MTT cytotoxicity assay

Both CCL-119 and WIL2-NS cells were seeded in a 96-well plate at a final concentration of 5 x 10^5^ cells/well and treated with 5 µM, 2.5 µM, 1.25 µM, 0.63 µM, 0.32 µM, and 0.16 µM of organotin (IV) dithiocarbamate compound for 24 hours at 37°C with 5% CO_2_. VCR was used as a positive control in this study, while cells without treatment were used as a negative control. After 24 hours of exposure, 20 µL of MTT (Merck) salt solution was added into each well and incubated for another 4 hours. The plate was wrapped with aluminium foil as MTT salt is light sensitive. After 4 hours, the purple crystal formazan was formed. All the medium was removed carefully from each well, and 200 µL DMSO was added to each well to dissolve the purple formazan crystal. Then, the plates were incubated for an additional 15 minutes. The results were analyzed and recorded using an ELISA microplate reader at 570 nm. The data was then plotted into a graph, and IC_50_ values were determined.

### 2.5. Selectivity index (SI)

The selectivity index (SI) is determined by dividing the cytotoxic activity (IC_50_ value) of each compound on a non-cancerous cell line (WIL2-NS) by the leukemia cell line (CCL-119). SI of more than 2 showed that the compound is more selective towards cancer cells than non-cancerous ones. WIL2- NS cell lines were selected as the comparator because it is a non-cancerous human B lymphoblast cell line, which is like CCL-119 (T-lymphoblastic leukemia), derived from lymphocytes. This choice ensures that comparisons are made between biologically similar cell types, minimizing variability that could arise from differences in tissue origin or lineage.

SI can be calculated by the formula below:


$$SI=\frac{{IC}_{50}fornon-cancerouscellline}{{IC}_{50}forcancerouscellline}\mathrm{\backslash ]}$$

### 2.6. Mode of cell death determination

The mode of cell death can be observed through the Annexin V-FITC/PI staining (BD, USA). The result of this test is either apoptosis or necrosis. The CCL-119 cells were seeded first into a sterile 6-well plate with a density of 5 x 10^5^ cells/mL, and the compound solution of organotin (IV) dithiocarbamate was pipetted into the 6-well plate based on the IC_50_ values obtained from the MTT assay. VCR was used as a positive control, while cells without any treatment were used as a negative control. Then, the plate was incubated for 24 hours. After the incubation, the cell suspension in each well was collected into different Eppendorf tubes. The tubes were then centrifuged for 5 minutes at 1500 rpm at 4°C. After the centrifuge, the supernatant in the tubes was removed, and the cell pellets were washed twice with cold PBS. Then, centrifuge the tubes again and remove the supernatant. Next, 100 µL of Annexin V binding buffer was resuspended in each tube, and 5 µL Annexin V-FITC was pipetted into the tube to stain the cells for 15 minutes at room temperature, dark. After that, 5 µL of propidium iodide was piped into the tube for 5 minutes. The staining process is done in dark conditions. Lastly, the sample was transferred into the Falcon tube, and each tube was resuspended in 400 µL of Annexin V binding buffer. The sample was analyzed using a flow cytometer (BD FACS Canto II) within one hour.

### 2.7. Cell morphology

CCL-119 cells were cultured in 6-well culture plates at 5 × 10^5^ cells/mL. The CCL-119 cells were treated with organotin (IV) dithiocarbamate compounds and VCR using the IC_50_ values obtained from the MTT assay. Then, all cells were incubated at 37°C with a 5% CO_2_ supply. After 24 hours of incubation, the cell’s morphological changes were observed under an inverted light microscope (OLYMPUS CKX41) at 40x magnification.

### 2.8. Cell cycle analysis (PI/RNase staining)

The cells were cultured and seeded in a 6-well plate at 1 x 10^6^ cells/mL. VCR was used as a positive control, and untreated cells were used as a negative control in this assay. The concentration of the compounds and VCR was followed by IC_50_ values that were taken from the MTT assay.

After 24 hours of incubation, all cells in the plate were transferred to the Eppendorf tube and centrifuged for 5 minutes at 4°C at 1500 rpm. After centrifuging, the supernatant was removed, and cells were washed with 500 µL cold PBS three times. The pellet was fixed by using 70% ethanol overnight at −20°C. Next, the samples were stained with 500 µL of PI/RNase staining buffer and incubated for 15 minutes. The samples were then transferred into Falcon tubes. The data was recorded using a flow cytometer and analyzed using ModFit LT software.

### 2.9. Alkaline comet assay

The CCL-119 was cultured and seeded in a 6-well plate at a 5 × 10^5^ cells/mL concentration. VCR was used as a positive control, and untreated cells were used as a negative control in this assay. The cells were incubated for 1 hour. After incubation, cells in each well were transferred to the Eppendorf tube to centrifuge for 5 minutes at 4°C by using a refrigerated microcentrifuge. The supernatant was removed, and the samples were washed with 1 mL of cold PBS. This process was repeated 3 times. After completion of the process, the supernatant was removed, and the pellet was left for a while on top of the ice while preparing the slide.

About 140 µL of normal melting agar (NMA) is pipetted on top of the fully frosted slide, and a cover slip is put on the agar for 5 minutes or until the agar fully solidifies. After that, the cover slip was removed, and 80 µL of low melting agar (LMA) was added to an Eppendorf tube containing the pellet of cells. The suspension was then transferred into a frosted slide with NMA agar. The cover slip was then put back on top of the frosted slide for 10 minutes or until dry. Next, the slide was transferred into a Coplin jar that contained lysis buffer solution and Triton X-100. The slides were then incubated at 4°C overnight. The slides were arranged on an electrophoresis tank filled with electrophoresis buffer for 20 minutes. Electrophoresis is done for another 20 minutes at 25 V with 300 mA. The slides were washed with a neutralization buffer solution 3 times at a 5-minute time interval. Finally, 30 µL of ethidium bromide (50 µg/mL) was added, and the slide was observed by using fluorescence microscopy (OLYMPUS BX51TR). CometScore software is used to analyze the DNA damage of the cells. The comet assay was performed under alkaline conditions (pH > 13) to detect both single- and double-strand DNA breaks.

### 2.10. Statistical analysis

All experimental data are presented as mean ± standard error of the mean (SEM) from three independent experiments (n = 3). Statistical comparisons between treatment groups were performed using one-way analysis of variance (ANOVA), followed by Tukey’s post-hoc test to identify significant differences among groups. A p-value of less than 0.05 (p < 0.05) was considered statistically significant. All statistical analyses were conducted using GraphPad Prism version 9.0 (GraphPad Software, USA).

## 3. Result

### 3.1. Cytotoxicity of organotin (IV) dithiocarbamate compounds on CCL-119 and WIL2-NS

**Fig 2:**
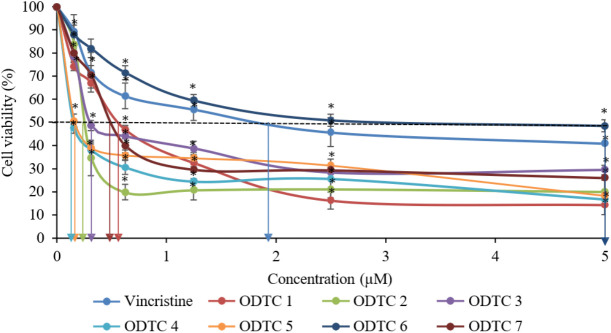
Cytotoxic effects of seven different organotin(IV) dithiocarbamate (ODTC 1–7) and vincristine towards CCL-119 cell line after 24 hours of exposure. The IC_50_ values obtained are as shown in the graph above. The data presented are the mean percentage of viable cells (%) ± SEM obtained from three different experiments (n = 3). *Significant difference (p < 0.05) from negative control.

MTT assay is a test that can identify cytotoxic effects of organotin (IV) dithiocarbamate compounds which are ODTC 1, ODTC 2, ODTC 3, ODTC 4, ODTC 5, ODTC 6, and ODTC 7, against CCL-119 (T lymphoblastic leukemia cells) and WIL2-NS (non-cancerous cells) by determining the percentage of cell viability after 24 hours of treatment. Through this assay, the median inhibitory concentration (IC_50_) value for each compound can be determined using the graph of the percentage of viable/living cells (%) against the concentration of the treatment compound. The concentration used in this assay is based on previous studies [13]. The treatment of organotin (IV) dithiocarbamate compounds and vincristine was able to cause cytotoxic and cytostatic effects on CCL-119 and WIL2-NS cells. From Fig 2, we can see that the treatment towards CCL-119 cells was able to produce an IC_50_ below 4 µM. ODTC 4 produced the lowest IC_50_ value (0.16 ± 3.22 µM), followed by ODTC 5 (0.19 ± 1.15 µM) and ODTC 2 (0.22 ± 0.04 µM). ODTC 6 produced the highest IC_50_ with a 3.10 ± 1.08 µM value. Meanwhile, the graph of cytotoxic effects of WIL2-NS treated with organotin (IV) dithiocarbamate compounds can be referred to from Rasli et al. (2023). We can see that the treatment was able to produce an IC_50_ below 5 µM. ODTC 7 produced the lowest IC_50_ (0.50 ± 0.34 µM), followed by ODTC 2 (0.52 ± 0.14 µM) and ODTC 4 (0.67 ± 0.14 µM). The treatment that produces the highest IC_50_ is ODTC 6 with a value of 5.00 ± 1.04 µM. Table 1 shows the summarized value of IC_50_ for leukemia cells (CCL-119) and non-cancerous cells (WIL2-NS).

**Table 1:** IC_50_ values and their selectivity index values for the seven organotin (IV) dithiocarbamate compounds and vincristine towards CCL-119 and WIL2-NS cells after 24 hours of exposure.

Compounds		IC_50_ (µM)			Selectivity Index (SI)
		CCL-119	WIL2-NS		
Diphenyltin		ODTC 1	0.56 ± 0.04	1.39 ± 0.46	2.48
	ODTC 2	0.22 ± 0.04	0.52 ± 0.14		2.36
Triphenyltin	ODTC 3	0.24 ± 2.00	0.75 ± 0.07		3.13
	ODTC 4	0.16 ± 3.22	0.67 ± 0.14		4.19
	ODTC 5	0.19 ± 1.15	0.79 ± 0.05		4.16
Dimethyltin	ODTC 6	3.10 ± 1.08	5.00 ± 1.04		1.61
	ODTC 7	0.50 ± 0.30	0.50 ± 0.34		1.00
Positive control	Vincristine (VCR)	1.82 ± 1.53	2.89 ± 0.03		1.59

### 3.2. Selectivity index (SI)

The selectivity index (SI) evaluates the selection characteristics of a treatment compound against cells. It was calculated by dividing the IC_50_ value of non-cancerous cells by the IC_50_ value of cancer cells. The selectivity index was evaluated based on the IC_50_ obtained from both cells for all compounds.

Table 1 shows that diphenyltin (IV) dithiocarbamate produces an index of selectivity of more than 2.00. As for triphenyltin (IV) dithiocarbamate, all the compounds produce a selectivity index of more than 3.00. This shows that the phenyltin (IV) dithiocarbamate compounds were selective towards leukemia cells (CCL-119) compared to non-cancerous cells (WIL2-NS). However, for dimethyltin (IV) dithiocarbamate compounds, both caused the general toxicity to both cells (SI < 2.00). The positive control used in this study also caused general toxicity (SI < 2.00).

### 3.3. Mode of cell death evaluation by using Annexin V FITC/PI staining

**Fig 3:**
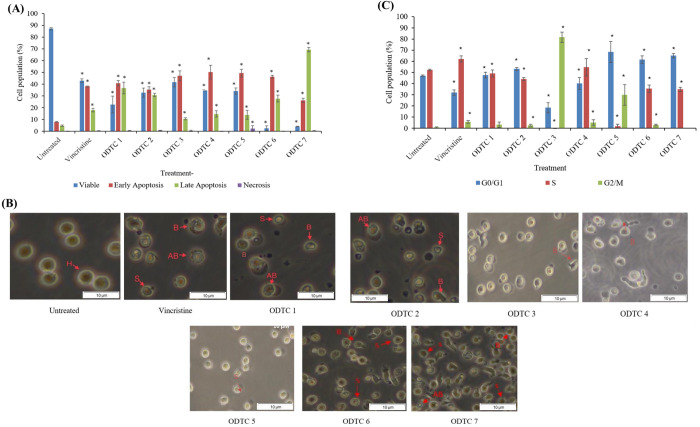
(A) Percentage of cell population treated with ODTC 1-7 compounds and Vincristine (VCR) after 24 hours of treatment. The data show the percentage of cells (%) ± SEM obtained from three individual experiments. (B) Morphological observation of CCL-119 cells after 24 hours of treatment with organotin (IV) dithiocarbamate compounds, VCR and untreated cells (magnification of 400×). Healthy cell (H), membrane blebbing (B), shrinkage cell (S), apoptotic body (AB). (C) Cell cycle arrest of organotin (IV) dithiocarbamate compound and VCR in CCL-119 cells. The effect of the treatment on cell cycle phase distribution is observed for 24 hours at the IC_50_ concentrations. A total of 15,000 events were acquired, and FACSDiva™ software and ModFit LT™ v3.0 were used to calculate the number of cells at the different phases. The data show the percentage mean (%) ± SEM obtained from three consecutive experiments. * Significant differences compared to negative control (p < 0.05).

There are many types of cell death. In this study, Annexin V FITC/PI was used to determine the type of cell death, either through apoptosis or necrosis. Annexin V stain will bind actively and specifically to phosphatidylserine (PS) residues, which can be found on a damaged phospholipid bilayer that undergoes the apoptosis process. Propidium iodide (PI) stain will enter the cells and bind to DNA due to the lysis of cell membranes. Thus, PI can detect cells that undergo necrosis or late apoptosis. Healthy cells with intact and normal phospholipid bilayers will not take up any stain and will appear double negative (Annexin V FITC negative, PI negative). Having established cytotoxic effects using the MTT assay, we next examined the mode of cell death via Annexin V FITC/PI staining. Fig 3 (A) shows the population of cell death upon treatment with seven organotin compounds (ODTC 1–7), and VCR after 24 hours of treatment. Generally, the treatment causes cell death through the apoptosis process with 77.6%, 66.3%, 57.7%, 65.1%, 63.4%, 74.2%, and 95.6%, respectively, for ODTC 1, ODTC 2, ODTC 3, ODTC 4, ODTC 5, ODTC 6, and ODTC 7. As for treatment with VCR also showed that the cells died through apoptosis (56.46%). From the graph, the population of cells undergoing necrosis is less than 1% for the treatment of organotin (IV) dithiocarbamate compounds and VCR (except ODTC 5, which is 4.37%).

### 3.4. Morphological observation

Morphological observation is done to support the data obtained from the mode of cell death evaluation using Annexin V FITC/PI stain. Fig 3 (B) shows morphological observation of CCL-119 cells after 24 hours of treatment with organotin (IV) dithiocarbamate compounds, VCR, and untreated cells by using IC_50_ values obtained from the MTT assay. This evaluation shows that the treatments caused apoptotic cell death characteristics such as cell membrane blebbing, cell shrinkage, and apoptotic body formation in CCL-119 cells. As for untreated cells, an intact round shape can be observed, indicating the healthy condition of the cells.

### 3.5. Cell cycle (PI/RNase staining)

There are three phases in the cell cycle: the G0/G1 phase, where cells are starting to grow; the S phase, where DNA in the cell is synthesised; and the G2/M phase, where cells continue dividing and growing. As shown in Fig 3 (C), the treatment of seven different organotin (IV) dithiocarbamate compounds causes cell cycle arrest at different phases. The treatment of ODTC 2, ODTC 6, and ODTC 7 caused the CCL-119 cell cycle to arrest at the G0/G1 phase. Treatment of ODTC 1 and ODTC 4 caused the cell cycle to arrest at the S phase. The treatment of ODTC 3 and ODTC 5 caused the CCL-119 cell cycle to arrest at the G2/M phase. The treatment of positive control towards CCL-119 causes the cell cycle to arrest at the S phase. Table 2 showed the summary of organotin (IV) dithiocarbamate effects on the cell cycle phases toward CCL-119 cell lines.

**Table 2:** Summary of organotin (IV) dithiocarbamate action on the cell cycle phases.

Compounds		Phases of Cell Cycle Arrest
Diphenyltin Triphenyltin	ODTC 1	S G0/G1 G2/M S G2/M
	ODTC 2	
	ODTC 3	
	ODTC 4	
	ODTC 5	
Dimethyltin	ODTC 6	G0/G1
	ODTC 7	G0/G1
Positive control	VCR	S

### 3.6. Alkaline comet assay

Alkaline comet assay is one of the methods that can be used to detect the genotoxic effect of a treatment on the DNA of cells. In this study, the genotoxic effects of organotin (IV) dithiocarbamate compound and VCR were evaluated in a treatment of CCL-119 cells for 1 hour of exposure.

**Fig 4:**
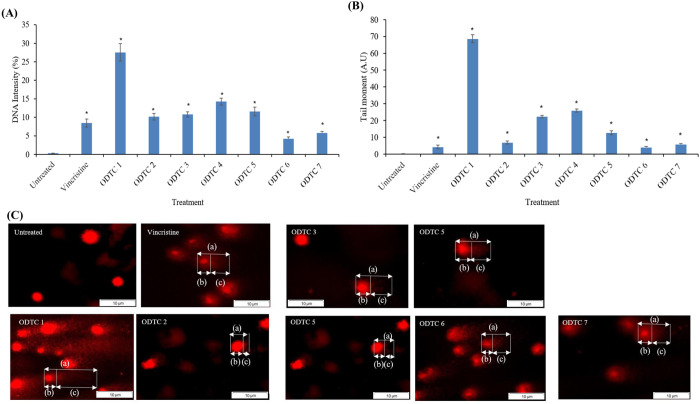
(A) Percentage of DNA intensity in the tail of CCL-119 cells after treatment with organotin (IV) dithiocarbamate compounds and vincristine compounds after 1 hour of exposure. (B) Tail moment of CCL-119 cells after treatment with organotin (IV) dithiocarbamate compounds and vincristine compounds after 1 hour of exposure. (C) Image of comet formation on CCL-119 cells treated with organotin(IV) dithiocarbamate compounds and vincristine after 1 hour of exposure. (a) Comet; (b) Head; (c) Tail. * Significant differences compared to negative control (p < 0.05).

Fig 4 (A) shows the percentage of DNA intensity in the tail of CCL-119 cells after treatment with all seven different organotin compounds and VCR after 1 hour of exposure. It showed significant differences between the treated cells compared to the untreated cells. CCL-119 cells treated with ODTC 1 compound have a higher DNA intensity (27.50 ± 2.36%, p = 0.00476) in the tail, followed by ODTC 4 (14.24 ± 0.94%) and ODTC 5 (11.59 ± 1.18%). The percentage of DNA intensity in the tail of CCL-119 cells treated with ODTC 2 and ODTC 3 is almost similar, with a value of 10.18 ± 0.89% and 10.79 ± 0.72%, respectively. ODTC 6 and ODTC 7 treatment caused the lowest percentage of DNA intensity in the tail (4.20 ± 0.56% and 5.75 ± 0.50%, respectively). As for treatment with VCR, the percentage of DNA intensity in the tail obtained is 8.48 ± 1.09%. Tail moment evaluation was done to measure the product of the tail length and the percentage of DNA in the tail. Fig 4 (B) shows the tail moment of the CCL-119 cell treated with organotin (IV) dithiocarbamate compounds and vincristine compounds after 1 hour of exposure. The analysis was done by using TriTek CometScore^TM^. The result showed that treatment with ODTC 1 caused the longest tail moment (68.58 ± 3.31 A.U). ODTC 4, ODTC 3 and ODTC 5 produced tail moments with values of 25.83 ± 2.60 A.U, 22.25 ± 0.93 A.U and 12.61 ± 1.56 A.U. ODTC 6, ODTC 7, ODTC 2 and vincristine caused the lowest tail moment with values of 3.90 ± 0.44 A.U, 5.69 ± 0.98 A.U, 6.83 ± 2.58 A.U and 4.17 ± 0.74 A.U, respectively. The comet formation on the nucleus cell of CCL-119 cells was caused after 1 hour of treatment with ODTC 1, ODTC 2, ODTC 3, ODTC 4, ODTC 5, ODTC 6, ODTC 7, and VCR, as shown in Fig 4 (C).

## 4. Discussion

Acute lymphoblastic leukemia (ALL) is a type of leukemia that commonly affects children worldwide. Currently, chemotherapy treatments, including vincristine as an anticancer drug, can cause side effects such as neurotoxicity, bone marrow suppression, and gastrointestinal distress in children with acute lymphoblastic leukemia. Therefore, new alternative treatments are needed to overcome this problem. In this study, the seven new organotin (IV) compounds, which are ODTC 1, ODTC 2, ODTC 3, ODTC 4, ODTC 5, ODTC 6, and ODTC 7, are evaluated for their potential as new chemotherapy agents for acute lymphoblastic leukemia cells (CCL-119). This evaluation involves screening their cytotoxic effects, identifying modes of cell death, assessing effects on cell cycle arrest, and investigating genotoxic effects.

This study utilized the MTT assay to evaluate the cytotoxic effects of organotin (IV) dithiocarbamate compounds. The percentage of cell viability was measured after 24 hours of treatment, with IC_50_ values calculated from the concentration-response relationship. ODTC 1-7 have different parent structures and functional groups, such as methyl and phenyl. The result proved that these compounds have a cytotoxic effect on CCL-119 cells with different degrees of toxicity. Previous studies indicate that various functional groups, including alkyl and aryl groups bonded to the tin atom, are thought to stimulate and exert a toxic effect on cancer cells [9]. All the compounds have different numbers of carbons (2 carbons, 3 carbons) or alkyl groups (ethyl and isopropyl) or allyl groups, which are capable of being toxic to cancer cells. According to Abd Aziz et al. (2023) [14], organotin compounds are toxic to cancer cells depending on the chemical structure present in the compounds. A previous study done by Pellerito et al. (2006) [8] showed that the activity of organotin (IV) compounds depends on their chemical structure. Meanwhile, the IC_50_ inhibitory value of the ODTC 2 compound was lower than the IC_50_ inhibitory value of the ODTC 1 compound after 24 hours of exposure. This shows that ODTC 2 compounds have a stronger toxicity effect than ODTC 1 compounds. ODTC 1 compounds have an isopropyl group with a strong electron-withdrawing feature, reducing the electron density on the S atom at C-S. Its ability to coordinate with the stannous atom shows that the bond of the isopropyl group with phenyl-titanium is weak. The ODTC 2 compound has an allyl group, which is an alkene group where the chemical structure has a double bond, which can make it more reactive [15]. Therefore, the ODTC 2 compound exerts a stronger reactivity effect and, hence, exerts a more toxic effect than the ODTC 1 compound against CCL-119 cells. Binding ligands in these compounds are crucial for inducing cytotoxic effects on cancer cells. The ligand used in this study, dithiocarbamate, is lipophilic, which enhances the transport of compounds across the cell membrane, acting as a carrier. It inhibits enzyme activity, disrupting cell biology and cytotoxic effects on CCL-119 cells. Dithiocarbamate ligands also have a strong ability to bind metals due to the presence of two sulfur (S) atoms in their molecular structure. As a result, these ligands can inhibit enzymes, disrupt biological activity or environments, and subsequently influence the cytotoxic effects on cells [16,17]. Kamaludin et al. (2017) [7] found that dithiocarbamate ligands’ lipophilicity inhibits enzymes and disrupts biological systems, causing toxicity in cancer cells. Adeyemi and Onwudiwe (2018) [6] demonstrated that enhanced lipophilicity boosts biological activities like antimicrobial and cytotoxic effects.

New treatments require chemotherapy agents that selectively target cancer cells without affecting non-cancerous cells. In this study, ODTC 6 and ODTC 7 compounds show non-selectivity towards CLL- 119 cells, with a selectivity index (SI) below 2, indicating toxicity to non-cancerous cells. In contrast, ODTC 1 to ODTC 5 compounds have SI values above 2, demonstrating greater selectivity for cancer cells. According to Badisa et al. (2009) [18], a higher SI indicates stronger selectivity for cancer cells, which is crucial for assessing anticancer drug effectiveness [19]. The positive control, vincristine, has an SI less than 2, as selective values are subjective; some studies consider SI above 1 as selective against target cells. Krzywik et al. (2020) [20] note that SI above 1 suggests greater drug efficacy against tumor cells than toxicity to normal cells, while many studies regard SI above 2 as selective [21]. Based on the MTT assay results, all ODTC compounds exhibited potent cytotoxicity against CCL-119 cells, with low IC_50_ values indicating strong antiproliferative activity. To explore the underlying mechanism of this cytotoxicity, we next examined the mode of cell death using Annexin V- FITC/PI staining and morphological observations. These analyses confirmed that all compounds induced apoptosis rather than necrosis. Apoptosis is a regulated and non-inflammatory form of cell death that is widely recognized as a desirable outcome for anticancer agents, as it allows for targeted elimination of malignant cells without damaging surrounding tissues [22]. Apoptosis can be detected by Annexin V, a recombinant protein that binds selectively and with high affinity to PS residues exposed on the outer leaflet of the plasma membrane during early apoptotic events [23]. Many previous studies indicate that organotin (IV) dithiocarbamate compounds lead to apoptosis. Our tests on Jurkat E6.1 cells demonstrate that all compounds induce early and late apoptosis except for dimethyltin (IV) diisopropyl dithiocarbamate [24]. Tabassum and Pettinari (2006) [8] reported that organotin compounds possess pro-apoptotic properties, largely due to the structural features of ODTC 1–7, which contain phenyl and methyl groups that may exhibit lipophilic characteristics. These traits enhance their solubility in lipid-rich environments, enabling effective interaction between the phenyl, methyl groups and PS residues [23,25]. Several earlier studies have demonstrated that organotin (IV) compounds typically induce cell death via apoptosis. For example, Awang et al. (2015) [26] reported that treatment with triphenyltin (IV) butylphenyl dithiocarbamate in Jurkat E6.1 cells resulted in a predominant induction of early-phase apoptotic cell death.

Morphological changes in CCL-119 cells treated with all seven organotin (IV) dithiocarbamate compounds indicate apoptosis. Key features include cell shrinkage, membrane blebbing, and apoptotic bodies. Apoptosis is preferred over necrosis, as necrosis causes inflammation due to membrane integrity loss and the release of cytoplasmic components [22].

A cell cycle assay identified cell cycle arrest at G0/G1, S, and G2/M phases. Compared to controls, compounds ODTC 2, ODTC 6, and ODTC 7 showed no cell proliferation at the G0/G1 phase. According to Pellerito et al. (2006) [8], organotin (IV) dithiocarbamate compounds, with functional alkyl and aryl groups, have anti-proliferative effects. Isopropyl and ethyl groups in ODTC 6 and ODTC 7 may contribute to toxicity against CCL-119 cells, causing G0/G1 arrest. Awang et al. (2011) [13] noted that these compounds could be developed as new chemotherapy agents due to their antiproliferative properties. ODTC 1 and ODTC 4 arrest the cell cycle at S phase, which was associated with significant DNA damage, suggesting that replication stress or interference with DNA synthesis may contribute to their cytotoxic mechanisms and the tumour suppressor protein p53 activation. Through cell cycle arrest or apoptosis, the molecular mechanism that activates p53 causes certain cytotoxic effects [27]. ODTC 3 and ODTC 5 induced G2/M phase arrest along with DNA damage, indicating activation of the G2/M checkpoint in response to DNA strand breaks, which likely ended in apoptotic cell death. These phase-specific disruptions in the cell cycle likely contribute to the activation of apoptotic pathways and accumulation of DNA damage, as observed in the comet assay results. The alkaline comet assay identified genotoxic effects in this study. DNA damage after 1-hour exposure to organotin (IV) dithiocarbamate compounds and VCR against CCL-119 leukemia cells was assessed using tail moment (A.U.) and DNA intensity (%). According to Sharif et al. (2007) [28], cells exhibit DNA damage when TM > 5 A.U. and TI > 10%. Results indicated that ODTC 6 and ODTC 7 showed no DNA damage within 1 hour, as the average tail moment was below 5 A.U. and DNA intensity below 10%. These compounds contain weakly bound dimethyl groups with tin atoms, explaining the lack of DNA damage. ODTC 1–5 compounds exhibit genotoxic effects on leukemia cells after 1 hour of exposure, indicated by tail moments over 5 A.U and DNA intensity above 10%. ODTC 1 shows maximum DNA damage (class 3), while ODTC 2 presents moderate damage (class 2). The differences likely arise from the allyl and isopropyl groups binding to dithiocarbamate and phenyltin ligands. ODTC 1 compounds are believed to induce apoptosis and DNA damage more effectively. This is likely due to the isopropyl group’s natural tendency to release electrons [13]. Compounds with fewer electrons can more readily bind with electron-rich molecules like DNA and proteins [6]. The isopropyl group facilitates electron removal from the diphenyltin compound, enhancing solubility and its binding to DNA, leading to damage. However, this study did not investigate whether the DNA damage observed is repairable or permanent, as we only observed it at 1 hour of exposure. Additional molecular assays, such as γH2AX staining and caspase or p53 expression analysis, are needed to further clarify the mechanisms of DNA damage and apoptosis induced by ODTC compounds.

All seven organotin (IV) dithiocarbamate compounds tested in this study induced apoptosis in CCL-119 acute lymphoblastic leukemia cells; however, they exhibited distinct cell cycle arrest patterns and variable genotoxic profiles. Notably, ODTC 2, ODTC 6, and ODTC 7 arrested cells in the G0/G1 phase, but only ODTC 2 caused detectable DNA damage. The absence of DNA damage in ODTC 6 and ODTC 7, despite induction of apoptosis, suggests a possible involvement of non-genotoxic pathways such as oxidative stress, mitochondrial dysfunction, or CDK inhibition. These findings imply that while apoptosis is a common endpoint, the upstream mechanisms differ, possibly due to structural variations among the compounds that affect subcellular targets or signalling cascades.

This mechanistic diversity highlights the importance of multi-assay approaches in evaluating novel anticancer agents. Compounds that induce apoptosis without detectable DNA damage may offer therapeutic advantages by minimizing genotoxic side effects, whereas those causing DNA damage might be more effective in highly proliferative or drug-resistant cancers. These findings provide a valuable foundation for future structure-activity relationship (SAR) studies and preclinical development of organotin-based chemotherapeutics.

## 5. Conclusion

All seven organotin (IV) dithiocarbamate compounds can induce cytotoxic effects, exhibiting lower IC_50_ inhibitory values against leukemia cells, CCL-119, and non-cancerous cells, WIL2-NS.

However, only ODTC 1–5 could produce selective effects (SI > 2.00) on CCL-119 leukemia cells. Evaluation of the mode of cell death using Annexin V FITC/PI staining indicates that all compounds lead to cell death through apoptosis. Morphological observations were conducted as a qualitative method to identify apoptotic cell death. Regarding cell cycle analysis, it was found that different compounds caused cells to arrest in various phases. The alkaline comet assay demonstrated that all compounds induced genotoxic effects at significantly different levels (p < 0.05) compared to the negative control. Further studies are needed to investigate the anti-leukemia properties of these compounds.

## 6. Limitations

Although the present findings demonstrate promising in vitro cytotoxic and pro-apoptotic effects of ODTC compounds on leukemia cells, further in vivo studies are required to evaluate their pharmacodynamics, systemic toxicity, and therapeutic efficacy in a physiological context. Additionally, the comet assay was performed following a short-term (1-hour) exposure, which reflects acute DNA damage. Longer or repeated exposures may reveal different genotoxicity patterns, including delayed or accumulative effects, and should be explored in future studies. Future studies using primary ALL samples or patient-derived xenograft models are also needed to confirm the in vivo relevance and therapeutic potential of ODTC compounds.

## Supporting information

S1 File Excel file containing raw data from CCL-119 cell line experiments. Sheet 1: MTT assay data showing cell viability after compound treatment. Sheet 2: Annexin V-FITC/PI apoptosis assay results. Sheet 3: Cell cycle distribution data from PI/RNase staining and flow cytometry. Sheet 4: Alkaline comet assay data assessing DNA damage.
(XLSX)
